# Predictive Biomarkers in Colorectal Cancer: From the Single Therapeutic Target to a Plethora of Options

**DOI:** 10.1155/2016/6896024

**Published:** 2016-08-01

**Authors:** Daniela Rodrigues, Adhemar Longatto-Filho, Sandra F. Martins

**Affiliations:** ^1^Life and Health Sciences Research Institute (ICVS), School of Health Sciences, University of Minho, Campus of Gualtar, 4710-057 Braga, Portugal; ^2^ICVS/3B's-PT Government Associate Laboratory, Braga/Guimarães, Campus of Gualtar, 4710-057 Braga, Portugal; ^3^Laboratory of Medical Investigation (LIM) 14, Faculty of Medicine, University of São Paulo, 01246-903 São Paulo, SP, Brazil; ^4^Molecular Oncology Research Centre, 14784-400 Barretos, SP, Brazil; ^5^Surgery Department, Hospitalar Center Trás-os-Montes e Alto Douro, Unidade Hospitalar de Chaves, Avenida Dr. Francisco Sá Carneiro, 5400-279 Chaves, Portugal

## Abstract

Colorectal cancer (CRC) is one of the most frequent cancers and is a leading cause of cancer death worldwide. Treatments used for CRC may include some combination of surgery, radiation therapy, chemotherapy, and targeted therapy. The current standard drugs used in chemotherapy are 5-fluorouracil and leucovorin in combination with irinotecan and/or oxaliplatin. Most recently, biologic agents have been proven to have therapeutic benefits in metastatic CRC alone or in association with standard chemotherapy. However, patients present different treatment responses, in terms of efficacy and toxicity; therefore, it is important to identify biological markers that can predict the response to therapy and help select patients that would benefit from specific regimens. In this paper, authors review CRC genetic markers that could be useful in predicting the sensitivity/resistance to chemotherapy.

## 1. Introduction

Colorectal cancer (CRC) is one of the most frequent cancers worldwide, being the third most frequent in men (10% of the total) and the second in women (9.2% of the total). Moreover, the mortality rate caused by CRC is the fourth highest in men (8% of the total) and the third in women (9% of the total) [1].

Several molecular mechanisms have been identified in CRC carcinogenesis, such as oncogenes activation, tumor suppressor genes inactivation [2], mutations in mismatch repair (MMR) genes, microsatellite instability (MSI) [3], and epigenetic alterations [4]. The accumulation of such alterations ultimately leads to neoplastic transformation.

The standard drugs for CRC chemotherapy are 5-fluorouracil (5-FU) and leucovorin (LV) in combination with irinotecan and/or oxaliplatin [5]. Current guidelines suggest the use of FOLFOX (5-FU/LV with oxaliplatin) or CapeOx (capecitabine and oxaliplatin) in stage III CRC, after surgical resection [6]. The MOSAIC (Multicenter International Study of Oxaliplatin/5-FU/LV in the Adjuvant Treatment of Colon Cancer) trial showed significant improvements in disease-free survival (DFS) and overall survival (OS) for FOLFOX compared with FL (5-year DFS: 73.3% versus 67.4%, resp., (*p* = 0.003) and 6-year OS 78.5% versus 76.0% (*p* = 0.046)) in stage III CRC but not in stage II [7]. The use of CapeOx regimen in the treatment of patients with stage III CRC showed an increase in 7-year OS when compared with those treated with 5-FU/LV (73% versus 67%; hazard ratio [HR]: 0.83; 95% confidence interval [CI]: 0.70–0.99; *p* = 0.04) [8]. In 2009, the PETACC-3 study investigated the efficacy of FOLFIRI (FU/LV with irinotecan) versus the 5-FU/LV regimen in patients with stage III CRC, with no significant differences in 5-year OS (73.6% versus 71.3% [*p* = NS]) and DFS (56.7% versus 54.3% [*p* = NS]). Therefore, regimens including irinotecan are not recommended in adjuvant therapy of stage III disease [9].

The use of adjuvant chemotherapy has been proven to increase OS in patients with stage III CRC but in stage II CRC this data remain controversial. That may suggest that the use of adjuvant chemotherapy has a greater advantage in people with higher risk [10]. Therefore, National Comprehensive Cancer Network (NCCN) guidelines recommend that treatment with 5-FU/LV, capecitabine, FOLFOX, CapeOx, or bolus 5-FU/LV/oxaliplatin (FLOX) should be considered in patients with stage II CRC and high risk of recurrence, defined as those with T4 tumors (stage IIB/IIC); poorly differentiated histology (exclusive of those cancers that are MSI-high [MSI-H]); lymphovascular invasion; bowel obstruction; lesions with localized perforation or close, indeterminate, or positive margins; or inadequately sampled nodes (<12 lymph nodes). In this setting, analyzing MSI plays a crucial role in the decision of whether to use adjuvant therapy in patients with stage II CRC [6]. This issue will be addressed later in this paper.

Metastatic CRC (mCRC) has been shown to benefit from neoadjuvant and adjuvant chemotherapy. A 2012 meta-analysis combining data from 3 studies and 642 patients showed an increase in DFS (pooled HR, 0.71; CI, 0.582–0.878; *p* = 0.001) and progression-free survival (PFS) (pooled HR, 0.75; CI, 0.620–0.910; *p* = 0.003) in CRC patients with liver metastasis treated with surgery plus chemotherapy, when compared to those treated with surgery alone. However, no increase on OS was observed (pooled HR, 0.743; CI, 0.527–1.045; *p* = 0.088) [11]. More recently, another meta-analysis showed similar results regarding perioperative chemotherapy in patients with resectable colorectal hepatic metastasis when compared to surgery alone. The data from 1896 patients from 10 studies showed a significant benefit in DFS in those who received perioperative therapy (HR, 0.81; 95% CI, 0.72–0.91; *p* = 0.0007) but no significant statistical difference in OS (HR, 0.88; 95% CI, 0.77–1.01; *p* = 0.07) [12].

According to current NCCN guidelines, one of the following regimens should be used in systemic chemotherapy of mCRC: FOLFOX, FOLFIRI, CapeOx, infusional 5-FU/LV or capecitabine, or FOLFOXIRI (FU/LV with oxaliplatin and irinotecan) [6]. Over the years, several studies were performed in order to evaluate the efficacy of one regimen over the others. In 2005, the GOIM (Gruppo Oncologico dell'Italia Meridionale) trial compared the efficacy of FOLFIRI versus FOLFOX regimens in 360 patients. The results showed no differences in OS (15 versus 14 months [*p* = NS]), response rate (RR) (31% versus 34% [*p* = NS]), and time to progression (TTP) (7 versus 7 months [*p* = NS]) [13]. Two other clinical trials tried to compare the efficacy and toxicity of FOLFIRI versus FOLFOXIRI regimens. Souglakos et al. found no differences in OS (19.5 versus 21.5 months [*p* = NS]), RR (34% versus 43% [*p* = NS]), and TTP (6.9 versus 8.4 months [*p* = NS]) [14]. On the other hand, Falcone et al. showed an improvement in RR (60% versus 34% [*p* < 0.0001]) and PFS (9.8 versus 6.9 months [*p* = 0.0006]) for the FOLFOXIRI group however at cost of increased toxicity (*p* < 0.001) [15]. A randomized trial compared the efficacy of CapeOx versus FOLFOX, with results showing no significant differences in PFS (8.0 versus 8.5 months, resp.) (HR, 1.04; 97.5% CI, 0.93–1.16) and OS (19.6 versus 19.8 months, resp.) (HR, 0.99; 97.5% CI, 0.88–1.12) [16].

Most recently, biologic agents such as cetuximab/panitumumab (monoclonal antibodies directed against the EGFR [epidermal growth factor receptor]) and bevacizumab (a humanized monoclonal antibody that targets VEGF [vascular endothelial growth factor]) have been proven to have therapeutic benefits in mCRC alone or in association with standard chemotherapy. In the CRYSTAL trial, Van Cutsem et al. investigated the efficacy of cetuximab plus FOLFIRI as first-line treatment for mCRC versus FOLFIRI alone in a total of 599 patients. There was an increase in PFS in the cetuximab-FOLFIRI group (HR, 0.85; 95% CI, 0.72–0.99; *p* = 0.048), but there was no difference in OS (HR, 0.93; 95% CI, 0.81–1.07; *p* = 0.31) [17]. Another study involving 813 patients with previously untreated mCRC compared the efficacy of bevacizumab along with irinotecan, bolus fluorouracil, and LV (IFL) versus IFL alone. Results showed an improvement in median survival (HR, 0.66 [*p* < 0.001]), PFS (HR, 0.54; *p* < 0.001), overall response rate (44.8% versus 34.8% [*p* = 0.004]), and median duration of relapse (HR, 0.62; *p* = 0.001) in the group treated with bevacizumab [18]. Therefore, the use of these targeted agents can be considered as initial therapy for mCRC, together with one of the regimens mentioned above [6].

Despite all the evidence and current recommendations, patients present different responses to treatment, regarding efficacy and toxicity. Therefore, it is important to identify biological markers that can predict the response to therapy and help select patients that would benefit from specific regimens [19].

In this paper, authors review CRC genetic markers that could be useful in predicting the sensitivity/resistance to chemotherapy.

## 2. 5-Fluorouracil

5-FU is the basis of CRC chemotherapy regimens. Its antitumor effect relies on the inhibition of thymidylate synthase (TS), the rate-limiting enzyme in the formation of thymidylate (TMP), which is essential for DNA synthesis. 5-FU enters cells and is converted to fluorodeoxyuridine monophosphate, which is the substrate to TS [20].

Studies have shown that tumors that express high levels of TS are associated with resistance to 5-FU [21, 22]. A study correlated the TS protein and gene expression with response to 5-FU based therapy in patients with CRC and gastric cancer. Patients with responsive disease had a mean TS protein level of 0.17 ± 0.03 arbitrary units (range, 0.05 to 0.38), whereas in patients whose tumors did not respond, the mean TS protein level was significantly higher, 0.60 ± 0.09 (range, 0.06 to 1.01; *p* < 0.01). Similarly, in patients with responsive disease, the mean TS : *β*-actin gene ratio was 1.36 ± 0.3 (range, 0.5–3.3). On the other hand, biopsies from patients with unresponsive disease had a mean TS : *β*-actin gene ratio of 15.4 ± 2.6 (range, 2.7–35.9; *p* < 0.01) [21]. A meta-analysis involving twenty studies tried to estimate the prognostic significance of TS expression in both advanced and localized CRC. The combined HR estimate for OS was 1.74 (95% CI, 1.34 to 2.26) and 1.35 (95% CI, 1.07 to 1.80) in the advanced and adjuvant settings, respectively. However, the authors assume possible existence of heterogeneity and publication bias [22].

The variability in TS expression seems to be related to polymorphisms in the TS promoter enhancer region (TSER), which determines TS expression. The two most common polymorphisms are the 3R and 2R variants. One study showed that mCRC with the 3R homozygous polymorphism (3R/3R) had higher levels of TS when compared with the 2R homozygous variant (2R/2R). In this trial, 50 patients with mCRC were treated with 5-FU. Individuals homozygous for the 3R variant had 3.6 times higher TS mRNA levels compared to those homozygous for the 2R variant in tumor tissue (*p* = 0.004). The authors then evaluated whether the TS polymorphism could predict the clinical outcome. The RR of individuals carrying the 2R/2R genotype was 50% against 9% of the 3R/3R patients (*p* = 0.041). Heterozygous individuals showed RR of 15%. Moreover, patients displaying the 3R homozygous polymorphism had less severe side effects to 5-FU (*p* = 0.008) [23].

However, there are 3R/3R variants that express low levels of TS. The cause appears to be a single nucleotide polymorphism (SNP) in the 3R allele, in which a guanine is substituted by a cytosine, resulting in low TS expression [24]. Tumors expressing this polymorphism could therefore benefit from therapy with 5-FU.

Another aspect that should be taken into account is the loss of heterozygosity (LOH) that has been observed in the TS locus of CRC. When LOH is present, in 2R/3R variant, it could originate either a 2R/loss or a 3R/loss tumor. This would ultimately affect its sensitivity to fluoropyrimidine-based regimens [25].

*Thymidine phosphorylase* (TP) is another enzyme involved in nucleotide metabolism. Concerning fluoropyrimidine therapy, TP is responsible for the conversion of 5′-deoxy-5-fluorouridine (5′-FUR) into 5-FU. TP has been proven to have a dual role in CRC: it is necessary for 5-FU prodrug activation and it has a role in promoting angiogenesis. Tsujitani et al. investigated the prognostic significance of microvessel density and the relationship between the expression of VEGF, TP, and angiogenesis in patients with gastric carcinoma. Their results revealed that tumors positive for VEGF and TP had high microvessel density, while VEGF and TP negative tumors showed low microvessel density. This evidence strongly suggests an association of TP with tumor neovascularization [26].

Even though high tumor TP expression leads to increased angiogenesis and therefore a poor prognosis (which is linked to increased infiltration, growth, and metastization) [27], it has been documented that it also increases sensitivity to 5-FU and thus its effectiveness in CRC treatment [28].

Tumor response to 5-FU therapy also depends on its bioavailability to ultimately inhibit DNA synthesis. DPD is the rate-limiting enzyme in 5-FU catabolism [29]; hence, high levels of DPD are linked with reduced fluoropyrimidine sensitivity, probably due to its decreased cytotoxic effect. Kornmann et al. showed that, among CRC patients treated with 5-FU, those who displayed low levels of DPD survived longer than those with high DPD tumors. They then concluded that DPD may have a prognostic value in CRC patients and those who would benefit the most from 5-FU based chemotherapy would be the ones who express the lowest levels of DPD [30]. Similarly, another study revealed that tumors from CRC patients who responded to therapy with 5-FU presented low levels of DPD [31].

Kornmann et al. tried to determine the prognostic value of TS and dihydropyrimidine dehydrogenase (DPD) in CRC patients receiving 5-FU. Resulting data showed that, among those treated with this drug, the ones who displayed higher levels of TS presented a better prognosis. Additionally, in each TS group, patients with low DPD survived longer than the ones with high DPD levels. Furthermore, the authors consider that the combination of TP and DPD is an independent prognostic factor for survival (*p* = 0.030) [30].

Nevertheless, individuals with DPD deficiency experience severe toxicity to 5-FU [32, 33]. Van Kuilenburg et al. performed a study, in which they evaluated the importance of DPD deficiency in 5-FU toxicity. They conducted a DPD activity, DPD gene, and clinical presentation analysis after 5-FU administration. Results demonstrated that 55% of patients with a decreased DPD activity suffered from grade IV neutropenia compared with 13% of patients with a normal DPD activity (*p* = 0.01). Moreover, toxicity was observed twice as fast in patients with low DPD activity as compared with patients with a normal DPD activity (10.0 ± 7.6 versus 19.1 ± 15.3 days; *p* < 0.05) [33].

## 3. Irinotecan

Irinotecan is an inhibitor of DNA topoisomerase I used in CRC treatment, usually in combination with 5-FU, as previously mentioned. It is a prodrug which is then converted to SN-38, the active metabolite that exerts both the antitumor and toxic effects [34]. SN-38 is then conjugated by UDP-glucuronosyltransferase 1A1 (UGT1A1) to SN-38G, an inactive metabolite [35]. UGT1A1 deficiency, caused by some polymorphisms, results in SN-38 accumulation, causing irinotecan-related toxicity, which includes diarrhea, dehydration and neutropenia.

The UGT1A1 enzyme is also involved in converting bilirubin into more soluble forms. Therefore, UGT1A1 deficiency can also lead to accumulation of unconjugated bilirubin, as seen in Crigler-Najjar and Gilbert syndromes [36, 37].

The UGT1A1 ^*∗*^28 polymorphism has been linked to severe side effects, namely, neutropenia and diarrhea. In this variant, the UGT1A1 expression is markedly decreased which leads to prolonged exposure to SN-38 [38]. In a 2014 meta-analysis, sixteen studies were included in order to investigate the association of UGT1A1 polymorphisms and irinotecan-induced diarrhea and neutropenia. UGT1A1 ^*∗*^28/ ^*∗*^28 genotype was associated with more than fourfold (OR, 4.79; 95% CI, 3.28–7.01; *p* < 0.00001) and threefold (OR, 3.44; 95% CI, 2.45–4.82; *p* < 0.00001) increases in the risk of neutropenia when compared with wild type and with at least one UGT1A1 ^*∗*^1 allele, respectively. Moreover, UGT1A1 ^*∗*^28/ ^*∗*^28 genotype was associated with a twofold risk of diarrhea (OR, 1.84; 95% CI, 1.24–2.72; *p* = 0.002). Additionally, the incidence of diarrhea was higher when irinotecan was given at higher doses (OR, 2.37; 95% CI, 1.39–4.04; *p* = 0.002) [37]. In 2005, the Food and Drug Administration (FDA) recommended that individuals homozygous for UGT1A1 ^*∗*^28 polymorphism would start the treatment with irinotecan with lower doses.

While UGT1A1 ^*∗*^28 is a common variant in both Caucasians and Asians, UGT1A1 ^*∗*^6 is another polymorphism that is only found in Asians and has similar toxic effects of those of the UGT1A1 ^*∗*^28 variant. A systematic review analyzed the association between UGT1A1 ^*∗*^6 polymorphisms and irinotecan-induced neutropenia and diarrhea in Asian patients. Patients with UGT1A1 ^*∗*^6/ ^*∗*^6 genotype displayed an increased risk for severe neutropenia (OR, 4.44; 95% CI; 2.42–8.14; *p* < 0.001). Individuals carrying the heterozygous variant also showed a higher risk for neutropenia, although not as high as the homozygous variant (OR, 1.98; 95% CI; 1.45–2.71; *p* < 0.001). UGT1A1 ^*∗*^6 homozygous patients were at higher risk for severe diarrhea (OR, 3.51; 95% CI; 1.41–8.73; *p* = 0.007), while heterozygous patients had no significant risk. Therefore, UGT1A1 ^*∗*^6 polymorphisms are potential biomarkers predicting irinotecan-induced severe toxicity in Asian patients. In this context, since 2008, Japan has been recommending the screening of patients for these two polymorphisms [39].

Even though genotyping for the UGT1A1 polymorphisms could be important in order to prevent severe adverse effects such as neutropenia, it does not predict response to chemotherapy.

## 4. Oxaliplatin

Oxaliplatin is a platinum analog chemotherapeutic agent with a 1,2-diaminocyclohexane (DACH) carrier ligand. It forms adducts on DNA strands, leading to DNA damage [26]. The nucleotide excision repair (NER) pathway is responsible for the removal of these adducts, reducing sensitivity to oxaliplatin. The two major genes involved in the NER pathway are excision repair cross-complementation group 1 (ERCC1) and xeroderma pigmentosa [27]. ERCC1 overexpression has been associated with resistance to oxaliplatin, probably because it induces repair of DNA strands. In 2001, Shirota et al. revealed that intratumoral ERCC1 mRNA expression levels are an independent predictive marker of survival for 5-FU and oxaliplatin combination chemotherapy in 5-FU-resistant mCRC. Patients with a low ERCC1 expression level had a greater median survival time than those with high levels of ERCC1 mRNA (10.2 versus 1.9 months, resp.; *p* < 0.001) [40]. A more recent study involving 180 stage III CRC patients treated with FOLFOX-4 showed that ERCC1 overexpression is an important predictor of early failure (*p* = 0.005), DFS (*p* <0.001), and OS (*p* < 0.001) [41]. However, conflicting data have been observed about the association of ERCC1 and clinical outcome in CRC patients treated with oxaliplatin. Li et al. conducted a trial to investigate the relationship between ERCC1 and DFS in Chinese CRC patients receiving oxaliplatin plus 5-FU. Results failed to show ERCC1 mRNA levels as a predictive factor for DFS (*p* = 0.638) [42]. Similar conclusions were achieved by Kim et al. in stage II and stage III CRC patients who underwent a curative resection followed by FOLFOX-4 adjuvant chemotherapy. ERCC1 expression was not significantly correlated with the 5-year DFS (*p* = 0.396) and therefore cannot be used as a prognostic factor [43].

Based on the inconsistent results concerning ERCC1 role in predicting patients' response to therapy, a meta-analysis was performed in order to elucidate the prognostic role of ERCC1 expression in patients with CRC. A total of 11 studies were analyzed. Results revealed that patients with higher ERCC1 expression showed a poorer OS (HR, 2.325; 95% CI; 1.720–3.143; *p* < 0.001), PFS (HR, 1.917; 95% CI; 1.366–2.691; *p* < 0.001), and response to chemotherapy (HR, 0.491; 95% CI; 0.243–0.990; *p* = 0.047), when compared to those with low ERCC1 expression. Therefore, the authors conclude that ERCC1 can indeed be a prognostic factor for OS, PFS, and response to chemotherapy [44]. However, further studies need to be addressed in order to better understand the true prognostic value of this biomarker.

Some studies showed that ERCC1-118 polymorphism influences response to oxaliplatin-based therapy. Furthermore, the allele ERCC1-118T induces higher levels of ERCC1 mRNA, which may explain why it is associated with shorter PFS in patients with mCRC treated with FOLFOX-4 regimen [45, 46]. This was also documented for XPD-751 polymorphism holding the C allele. Moreover, the combination of ERCC1-118 T/T genotype with either XPD-751 A/C or XPD-751 C/C resulted in an even shorter PFS (HR, 2.84; 95% CI, 1.47–0.45; *p* = 0.002), compared to the case when only one of these genotypes was present [46]. More recently, another study showed that the XPG Arg1104His polymorphism variants were associated with a longer DFS in patients receiving oxaliplatin-based chemotherapy [47]. Based on current data, ERCC1 screening is not recommended to select patients for oxaliplatin-based regimens.

## 5. Antibodies Targeting EGFR

The activation of EGFR induces cell proliferation by activating two main downstream signaling pathways, the Ras/Raf/mitogen-activated protein kinase (MAPK) (mainly responsible for cell proliferation) and the PI3KPTEN-AKT (mainly responsible for cell survivor) pathways.

The monoclonal antibodies cetuximab and panitumumab are directed against EGFR, therefore inhibiting cell proliferation and tumor growth, by binding to the extracellular domain of EGFR and blocking ligand-induced receptor phosphorylation and further signaling [48]. Response to EGFR-targeted therapy varies greatly between individuals, and it is not correlated with EGFR levels. A clinical trial conducted by Cunningham et al. showed that the degree of EGFR expression, either as the percentage of EGFR-positive tumor cells or as the maximal staining intensity per cell, had no relation to the clinical response rate (*p* = 0.67 and *p* = 0.84, resp.) [49]. Another study, designed to access the antitumor activity and toxicity of cetuximab in patients whose tumors express the EGFR, displayed similar results. Moreover, 88% of the patients presented skin reactions, such as acne-like rash and paronychia cracking, as well as allergic reactions, which in two cases led to therapy cessation. The authors also verified a statistical correlation between the presence and severity of the acne-like rash and survival (*p* = 0.02) [50]. Given the possible adverse effects of cetuximab, it is important to be able to select the patients that would benefit from therapy with this agent. Once scientific evidence showed no correlation between EGFR expression and clinical response, it was hypothesized that gene mutations in downstream proteins could be responsible for cetuximab/panitumumab resistance.

KRAS has been widely studied in the context of cetuximab/panitumumab response in mCRC. Several studies showed that KRAS mutations are highly predictive of cetuximab resistance in patients with mCRC. The most common KRAS mutations occur at exon-2 (codons 12 and 13), although those have also been found in exon-3 and exon-4 [51–53].

As previously mentioned, the CRYSTAL trial was conducted to investigate the efficacy of cetuximab plus irinotecan, 5-FU, and LV (FOLFIRI) as first-line treatment for mCRC [17]. The results showed improvement in PFS but not in OS in patients treated with cetuximab. However, in a subset analysis of patients with wild-type KRAS, the addition of cetuximab to FOLFIRI resulted in significant improvements in OS (median, 23.5 versus 20.0 months; HR = 0.796; *p* = 0.0093), PFS (median, 9.9 versus 8.4 months; HR = 0.696; *p* = 0.0012), and RR (57.3% versus 39.7%; OR = 2.069; *p* = 0.001) compared with FOLFIRI alone. No significant difference in efficacy was evident in patients with mutant KRAS (codons 12 and 13, exon-2) [17, 54]. Most recently, a revision of the CRYSTAL study showed that not only the mutations in codons 12 or 13 were linked to worst response to cetuximab plus FOLFIRI, but also the same was true to mutations in exon-3 and exon-4. DNA samples from CRYSTAL patients that presented a KRAS wild-type status for mutations in codons 12 and 13 were reanalyzed for mutations in four additional KRAS codons (exon-3 and exon-4) and six NRAS codons (exon-2, exon-3, and exon-4). Results showed no differences in OS and PFS between patients treated with cetuximab plus FOLFIRI and FOLFIRI alone [55].

Three important studies were conducted in order to assess the efficacy of FOLFOX plus cetuximab versus FOLFOX alone. In the OPUS (Oxaliplatin and Cetuximab in First-Line Treatment of Metastatic Colorectal Cancer) study, Bokemeyer et al. showed that the addition of cetuximab to FOLFOX-4, as first-line therapy in patients with mCRC, resulted in better response to chemotherapy (OR = 2.551; *p* = 0.0027) and PFS (HR = 0.567; *p* = 0.0064) in wild-type KRAS individuals when compared with those treated with FOLFOX-4 alone. Those who held the KRAS exon-2 mutations showed no benefit from cetuximab, as they presented with worse response to treatment and shorter PFS [56]. Another study called COIN also compared the efficacy of FOLFOX plus cetuximab versus FOLFOX alone in 1630 patients. In this trial, Maughan et al. showed that FOLFOX with cetuximab increased RR compared with FOLFOX alone (64% versus 57%, resp.; *p* = 0.049). However, no evidence of improved PFS (8.6 versus 8.6 months, resp.; HR = 0.96, 95% CI = 0.82–1.12, and *p* = 0.60) or OS (17.0 versus 17.9 months; HR = 1.04, 95% CI = 0.87–1.23, and *p* = 0.67) was seen among patients with wild-type KRAS. Furthermore, skin and gastrointestinal toxic effects were more frequent in the group treated with FOLFOX plus cetuximab compared to control group (14 versus 114 and 67 versus 97, resp.) in patients with KRAS wild-type tumors [57].

A multicenter phase III trial named NORDIC-VII investigated the efficacy of cetuximab plus a bolus of fluorouracil/folinic acid and oxaliplatin (Nordic FLOX), administered continuously or intermittently, in 571 patients with previously untreated mCRC. Therefore, the patients were randomly divided into three groups, one receiving standard Nord FLOX (arm A), another receiving FLOX plus cetuximab (arm B), and a third group treated with cetuximab plus intermittent FLOX (arm C). OS, PFS, and RR were similar in the 3 treatment arms (OS = 20.4, 19.7, and 20.3 months, resp. [*p* = NS]; PFS = 7.9, 8.3, and 7.3 months [*p* = NS]; and RR: 41%, 49%, and 47% [*p* = NS]). In KRAS wild-type tumors, the addition of cetuximab did not bring about additional benefits. In KRAS mutant tumors, improvement in PFS was reported in arm B for patients treated with cetuximab when compared with those receiving FLOX alone. However, this difference was not statistically significant (7.8 versus 9.2 months; HR = 0.71; *p* = 0.07) [58]. Based on these studies, the screening of patients with mCRC to exon-2 KRAS mutations is currently recommended. However, 65% of the wild-type patients for the exon-2 mutation are resistant to cetuximab, which brings up the importance of using new biomarkers such as exon-3 and exon-4 mutations [59].

KRAS has also been studied in the setting of panitumumab use in patients with chemotherapy-refractory mCRC. Also in this case, only the KRAS wild-type patients showed sensitivity to monotherapy with panitumumab. Amado et al. conducted a phase III trial, in which they compared the efficacy of panitumumab as monotherapy for mCRC to best supportive care (BSC). Additionally, the authors of this study tried to evaluate whether the efficacy of panitumumab was related to KRAS status. Results showed that, in those treated with panitumumab, PFS was significantly increased (HR, 0.45; 95% CI, 0.34–0.59; *p* < 0.0001) in the wild-type KRAS group compared to KRAS mutant group (HR, 0.99; 95% CI, 0.73–1.36). In patients with KRAS wild-type mCRC tumors, treatment with panitumumab resulted in higher PFS (HR, 0.45; 95% CI, 0.34–0.59; median PFS of 12.3 weeks for panitumumab versus 7.3 weeks for BSC). Contrarily, in the KRAS mutant group, no improvement in PFS was observed with therapy with panitumumab (HR, 0.99; 95% CI, 0.73–1.36; median PFS of 7.4 weeks for panitumumab versus 7.3 weeks for BSC) [60]. The phase III PRIME trial compared the efficacy and safety of FOLFOX-4 versus FOLFOX-4 plus panitumumab in mCRC patients with no previous chemotherapy treatment. Data from 1183 patients was collected. Among patients with KRAS wild-type tumors, results displayed a significant improvement in PFS in the group treated with FOLFOX-4 and panitumumab, when compared to the FOLFOX-4 group (9.6 versus 8.0 months, resp.; HR, 0.80; 95% CI, 0.66–0.97; *p* = 0.02). No differences in OS were observed (23.9 versus 19.7 months, resp.; HR, 0.83; 95% CI, 0.67–1.02; *p* = 0.072). On the other hand, in the mutant KRAS stratum, there was a significant decrease in PFS in the FOLFOX-4 plus panitumumab group (HR, 1.29; 95% CI, 1.04–1.62; *p* = 0.02). Median OS was 15.5 months versus 19.3 months, respectively (HR, 1.24; 95% CI, 0.98–1.57; *p* = 0.068) [61]. Therefore, patients who are candidates to therapy with panitumumab are indicated for screening for KRAS codons 12 and 13 mutations [60].

BRAF is an oncogene that encodes a downstream effector of KRAS in the MAPK pathway. Mutations in this gene may explain some of the cases in which KRAS wild-type patients do not respond to cetuximab/panitumumab therapy. The most common mutation found on BRAF is V600E, which has been shown to induce resistance to EGFR-targeted therapy. In a retrospective study, Di Nicolantonio et al. analyzed tumor responses, PFS, OS, and the mutational status of KRAS and BRAF in 113 tumors from cetuximab- or panitumumab-treated mCRC patients [62]. Data showed decreased PFS (*p* = 0.011) and OS (*p* < 0.0001) in patients with BRAF mutations. Furthermore, none of the patients that displayed the BRAF mutation responded to therapy and none of the responders carried this mutation (*p* = 0.029). This evidence suggested that the introduction of the V600E allele impaired the therapeutic effect of cetuximab/panitumumab. Therefore, the authors hypothesized that a BRAF inhibitor could restore the sensitivity to these drugs. Sorafenib, a clinically approved small-molecule kinase inhibitor, was then used to pharmacologically target BRAF. Results showed that sorafenib restored sensitivity to panitumumab or cetuximab of CRC cells carrying the V600E allele [62]. Additionally, KRAS and BRAF mutations have been shown to be mutually exclusive, with none of the KRAS-mutated samples carrying concomitant BRAF mutations, or vice versa. This evidence had been previously displayed in other studies [62–64].

Another retrospective study evaluated the effect of KRAS downstream mutations on the efficacy of cetuximab in 1022 patients with chemotherapy-refractory mCRC treated with cetuximab plus chemotherapy. Regarding the BRAF status, BRAF mutants had a significantly lower RR (8.3% [2/24] versus 38.0 [124/326] for wild types; OR, 0.15; 95% CI, 0.02–0.51; *p* = 0.0012) and disease-control rate (37.5% [9/24] versus 77.3% [252/326]; OR, 0.176; 0.071–0.41; *p* < 0.0001) and shorter PFS (median, 8 versus 26 weeks in wild types; HR, 3.74; 95% CI, 2.44–5.75; *p* < 0.0001) and OS (median, 26 versus 54 weeks in wild types, HR, 3.03; 1.98–4.63; *p* < 0.0001), when compared to wild types [65]. Although some retrospective studies showed a decreased response to cetuximab/panitumumab in patients with BRAF mutations, some patients may have some benefit from therapy with cetuximab as a front-line therapy [54, 66].

Therefore, the role of BRAF mutation in predicting response to EGFR-targeting agents is still unclear. On the other hand, its utility as a prognostic factor is more consensual [54, 67]. Van Cutsem et al. showed that BRAF V600E mutation was linked to a worse prognosis in KRAS wild-type patients, regardless of which treatment they were submitted to (cetuximab plus FOLFIRI versus FOLFIRI alone) [54]. Additionally, the above-mentioned COIN trial revealed that, irrespective of treatment received, OS was shorter in patients whose tumors had mutations in BRAF (*n* = 102, 8.8 months, IQR: 4.5–16.1) than in those with BRAF wild-type tumors. This data confirms once again that BRAF mutation is an indicator of poor prognosis [57].

Despite the still controversial role of this downstream mutation as a predictive marker, the NCCN panel recommends the BRAF genotyping for stage IV CRC, at the time of diagnosis. They consider the response to cetuximab/panitumumab in BRAF mutant mCRC to be highly unlikely [6].

PTEN is a member of the EGFR signaling pathway, PI3K-PTEN-AKT. In 2007, Frattini et al. showed that loss of PTEN expression was predictive of cetuximab resistance in mCRC patients. In a study involving 27 patients, 11 presented loss of PTEN activity, and none of those responded to therapy with cetuximab (*p* < 0.001). Furthermore, patients who responded to cetuximab presented with PTEN expression and wild-type KRAS genotype [68].

PIK3CA mutations were also linked with resistance to EGFR-targeted therapy [69, 70]. However, the role of PIEK/PTEN on predicting response to cetuximab/panitumumab is still controversial. A recent study showed that neither PIK3CA mutations nor PTEN expression levels would predict mCRC patients' response to cetuximab [71].

Besides the molecules that integrate the EGFR signaling pathways, molecules that act in an autocrine or paracrine way may play a role in predicting the response to cetuximab/panitumumab [72]. Amphiregulin and epiregulin are EGFR ligands whose increased expression has been linked to a good response to EGFR-targeted therapy [73, 74].

## 6. Bevacizumab

Bevacizumab is a monoclonal antibody that targets VEGF-A and it is used in the treatment of mCRC in addition to chemotherapy. 18 VEGF-A is a proangiogenic ligand which (together with other proangiogenic molecules) has been associated with tumor vascularization, progression, and metastization. Moreover, it is known to blunt the cytotoxic effect of chemotherapy by recruiting endothelial cells, protecting the tumor from chemotherapy. Bevacizumab blocks this process, by “normalizing” tumor's vascularization and allowing chemotherapy and oxygen to reach tumor cells [75]. Studies have been made in order to identify biomarkers that could predict patients' response to bevacizumab. High VEGF-A levels have been associated with a poor clinical outcome. However, they do not predict patients' response to bevacizumab, as shown by an initial comprehensive analysis of plasma VEGF-A as a biomarker across multiple phase III trials, which included the AVF2107 study [76]. In another study, Hayashi et al. showed that an early increase in VEGF-A circulating levels, after an initial decrease (in patients treated with bevacizumab plus FOLFIRI), was associated with a reduced PFS (*p* = 0.009) [77].

A 2013 study evaluated the predictive and prognostic value of VEGF using samples from HORIZON II and III trials, in which CRC patients were treated with cediranib, a VEGF receptor (VEGFR) tyrosine kinase inhibitor. The resulting data was consistent with previous studies, with high levels of VEGF associated with worse prognosis, independent of treatment received (HORIZON II OS: HR, 1.35; 95% CI, 1.12–1.63; HORIZON III OS: HR, 1.32; 95% CI, 1.12–1.54). However, baseline VEGF was not predictive of response to cediranib [78].

Although several studies linked high VEGF levels to a poor clinical outcome, most failed to demonstrate its value as a predictive biomarker for chemotherapy response. Given the importance of finding markers that would help select candidates for treatment with bevacizumab, Bruhn et al. conducted a trial, in which they determined tumor expression levels of the proangiogenic proteins (IL-6, IL-8, b-FGF, PDGF-BB, and VEGF-A) in mCRC patients. Individuals were divided into two groups (high or low), according to their levels of protein expression. Final data showed that low VEGF-A levels were linked to a better RR for bevacizumab (RR [low] 53% versus [high] 19%, interaction *p* = 0.03), while “high” VEGF-A was prognostic for shorter PFS (unadjusted HR: 1.34, *p* = 0.06; adjusted HR: 1.55, *p* = 0.008). The other proangiogenic molecules showed neither a prognostic nor a predictive value [79].

More recently, Tsai et al. performed a clinical trial to compare pre- and posttherapeutic VEGF immunohistochemical (IHC) expression in 57 mCRC patients treated with FOLFIRI plus bevacizumab. Results showed that low posttherapeutic VEGF expression (*p* < 0.001) and decreased peritherapeutic VEGF expression (*p* < 0.001) were significantly predictive factors for therapy response. Moreover, patients with decreased peritherapeutic VEGF expression had improved 6-month PFS than those with no peritherapeutic VEGF alterations (*p* = 0.033) [80]. Given the inconsistent data regarding the role of VEGF in response to therapy, its use as a predictive biomarker for treatment with bevacizumab is currently not advised. Besides VEGF-A, several other molecules play a role in angiogenesis, some of which have been the object of clinical studies in order to identify biomarkers of bevacizumab resistance. Although some of them have shown a promising role as predictive markers, there is yet not enough data to support their use in the clinical practice [77, 81–83]. Further investigation is needed in the setting of mCRC patients' sensitivity/resistance to therapy with bevacizumab.

## 7. Microsatellite Instability

Microsatellites are short tandem DNA repeats that occur throughout the genome. When mutations in microsatellites occur in MMR genes (MSH2, MLH1, MSH6, and PMS2), allowing DNA replication errors to occur, they give rise to a defective MMR (dMMR) system. This is usually measured by either the presence of MSI or the absence of the protein products for the MMR genes. MSI tumors can be classified as MSI-H or MSI-low (MSI-L), depending on the extent of instability in the marker tested. Tumors that do not present MSI are called microsatellite-stable (MSS). MSI-H occur in about 15% of CRC (in this case, the hypermethylation of the MLH1 gene promoter inactivates the mismatch repair), particularly in stage II CRC. MSI-H are characterized by a proximal colon predominance, older age, and female sex [84, 85].

MSI tumors have been linked to a good prognosis and resistance to fluoropyrimidine-based chemotherapy alone. In 2003, Ribic et al. investigated the usefulness of MSI status as a predictor of the benefit of adjuvant chemotherapy with fluorouracil in stage II and stage III colon cancer. 570 patients from five previous randomized trials who had been randomly assigned to receive adjuvant 5-FU based chemotherapy after surgical resection or no treatment were studied. Results showed that 5-FU based therapy benefited those with MSS or MSI-L tumors but not those who presented a MIS-H phenotype. Patients with MSS who had MSI-L tumors and were submitted to adjuvant therapy showed improved OS (HR, 0.72; 95% CI, 0.53–0.99; *p* = 0.04). By contrast, MSI-H tumor patients did not benefit from adjuvant chemotherapy. Patients who did not receive adjuvant therapy and presented MSI-H tumors showed a better five-year rate of OS than patients with tumors exhibiting a MSS or MSI-L status (HR, 0.31; 95% CI, 0.14–0.72; *p* = 0.004) [86].

In a similar study, Sargent et al. examined the MMR status as a predictor of adjuvant 5-FU based therapy benefit in patients with stages II and III colon cancer. As in the previous study, a group of 457 patients were randomly assigned to receive either 5-FU based adjuvant therapy or no postsurgical treatment. Patients with MSS tumors who received adjuvant therapy showed a significantly improvement in DFS (HR, 0.67; 95% CI, 0.48–0.93; *p* = 0.02). On the other hand, defective MMR (dMMR) tumor patients showed no benefit from 5-FU based therapy when compared with those treated with surgery alone (HR, 1.10; 95% CI, 0.42–2.91; *p* = 0.85). In patients with stage II, dMMR CRC, treatment was associated with reduced OS (HR, 2.95; 95% CI, 1.02–8.54; *p* = 0.04) [87].

As mentioned above, MSI tumors are associated with a good prognosis. Data from the PETACC-3 study showed that the MSI-H profile occurs more often in stage II CRC than in stage III (22% versus 12%, resp.; *p* < 0.0001) [88]. Furthermore, evidence showed that these tumors may have a decreased likelihood to metastasize. Koopman et al. collected data from a phase III study in 820 advanced CRC patients, in which dMMR was only found in 18 (3.5%) patients [85].

All these evidences suggest that stage II CRC with a MSI-H profile may have good prognosis and would not benefit from adjuvant 5-FU based chemotherapy. On the other hand, in stage III disease, the benefit of adjuvant 5-FU based chemotherapy is well documented. Flejou et al. studied the benefit from adding oxaliplatin to a 5-FU based regimen in stage III CRC, using data from MOSAIC study patients.

This report had previously shown that combining oxaliplatin with 5-FU and LV improves OS and DFS, as mentioned before [7]. After analyzing the MMR status of 986 patients, Flejou and his colleagues displayed data that supports the use of FOLFOX-4 regimen in stage III CRC with a MSI-H profile. HR for stage III dMMR CRC are 0.56 (0.19–1.61) for RFS, 0.51 (0.18–1.41) for DFS, and 0.44 (0.15–1.34) for OS, respectively. HR for stage II dMMR tumors are 0.64 (0.11–3.70) for RFS, 0.60 (0.17–2.09) for DFS, and 0.52 (0.13–2.10) for OS, respectively [89].

While stage III CRC should be treated with 5-FU plus oxaliplatin, stage II CRC treatment is more controversial, as these patients do not benefit as much from adjuvant therapy [62]. Patients with stage II disease often display good prognosis and have been shown to be resistant to 5-FU based regimens. Therefore, the NCCN currently recommends determining MSI status for all patients with stage II CRC, and adjuvant treatment should not be given to patients with low-risk MSI-H tumors [6].

Despite all these studies and NCCN recommendations, a recent meta-analysis concluded that there are no differences in the effect of 5-FU based chemotherapy, regarding the MSI status.

Although there was improvement in DFS (HR, 0.62; 95% CI, 0.54–0.71) and OS (HR, 0.65; 95% CI, 0.54–0.79) in patients with MSS tumors treated with 5-FU compared with those who were untreated and no improvement in the MSI group (DFS [HR, 0.84; 95% CI, 0.53–1.32] and OS [HR, 0.66; 95% CI, 0.43–1.03]), the effect of adjuvant therapy was not different at a statistical significant level [90].

Summary of potential predictive biomarkers, available data, and current recommendations can be observed in Table 1.

## 8. Discussion

CRC is one of the most frequent and deadliest cancers worldwide [1]. Systemic chemotherapy in the adjuvant and advanced setting has evolved greatly in the last decades from basic cytotoxic agents to combination regimens and, more recently, the advent of biological agents [5]. However, patients often present different responses and grades of toxicity to each regimen. Therefore, it is important to identify biomarkers that could predict response to treatment, thus helping us in selecting the best candidates for each drug. In this paper, we reviewed the main molecular markers that have been studied in the setting of CRC.

Studies regarding EGFR-targeting agents cetuximab and panitumumab have so far presented the most consistent results. As discussed before, current guidelines recommend screening patients for KRAS exon-2 mutations who are considered for cetuximab/panitumumab treatment. Wild-type KRAS patients benefit from improvement in PFS, OS, and RR with cetuximab, as shown in CRYSTAL and OPUS trials [17, 56]. In contrast, individuals who presented KRAS mutation had shorter PFS and OS. However, COIN and NORDIC-VII failed to show association between KRAS status and response to therapy with cetuximab [57, 58].

In the PRIME trial, evidence showed that wild-type individuals who received panitumumab displayed better PFS, while those with mutant KRAS had no profit from this agent [61].

Therefore, we hypothesized that, in those cases in which KRAS wild-type patients appeared to be resistant to EGFR-targeting agents, other unidentified mutations may be present and may be the cause for the lack of response. Thus, further studies should be addressed in order to investigate the role of KRAS exon-3 and exon-4 mutations as predictive biomarkers for therapy with cetuximab/panitumumab.

BRAF V600E mutation has shown some prognostic value in CRC patients. Nevertheless, its predictive value is still not established, as current studies show inconsistent results. Still, the NCCN recommends the screening for BRAF mutations before initiating treatment with cetuximab/panitumumab [6].

Even though current data strongly supports the use of chemotherapeutic agents in stage III and stage IV CRC, the use of adjuvant treatment in stage II disease is still controversial. In this setting, MSI status has been studied as a predictor of the benefit of adjuvant chemotherapy. Tumors displaying MSI-H phenotype have been shown to have better prognosis, as demonstrated in the PETACC-3 study [88]. They also appear to have a lower likelihood to metastasize. Moreover, scientific evidence established a predictive role for the MSI status. In fact, MSI-H tumors seem to have no benefit from 5-FU based chemotherapy [86, 87]. On the other hand, in stage III CRC patients who were submitted to adjuvant chemotherapy, MSI-H tumors have shown improvement in DFS and OS [89]. So it seems that low-risk stage II CRC, as are the ones that present MSI-H status, should not be submitted to cytotoxic therapy. Conversely, high-risk stage II CRC may have a clinical behavior closer to that of stage III disease and so would benefit from adjuvant chemotherapy. Therefore, the determination of the MSI status in stage II CRC patients is recommended to assess whether they are candidates to receive adjuvant treatment.

Irinotecan has been linked to severe toxicity, particularly in patients with a UGT1A1 deficiency.

The UGT1A1 ^*∗*^28 polymorphism seems to be responsible for low UGT1A1 expression levels and thus a higher frequency and severity of side effects. Moreover, in the Asian population, the UGT1A1 ^*∗*^6 variant was also identified and was associated with severe neutropenia and diarrhea [37]. Hence, recommendations advise the screening of all patients for the UGT1A1 ^*∗*^28 and UGT1A1 ^*∗*^6 (in Japan) variants before initiating treatment with irinotecan [39]. Although these polymorphisms can help predict the occurrence of severe toxicity, they are not predictors of therapy response.

So far, none of the other markers have proven a predictive value to therapy response. Studies concerning ERCC1 have shown contradictory results. Regarding the anti-VEGFR agent bevacizumab, data revealed that high VEGF-A levels are associated with poor prognosis.

However, studies addressing the role of VEGF as a predictive biomarker have shown inconsistent results. Thus, VEGF is not currently considered a useful marker of response to bevacizumab. In the future, studies addressing the role of other proangiogenic molecules should be conducted. Several angiogenesis-related factors, such as IL-8, soluble angiopoietin II (sANG-2), basic FGF (b-FGF), and stem cell factor (SCF), have been shown to be increased in CRC patients that did not respond to bevacizumab. However, the available data is still insufficient [77].

## 9. Conclusion

In this paper, the authors reviewed the role of actual predictive biomarkers in CRC. KRAS is the best studied marker of response to cetuximab/panitumumab, with current use in clinical practice. Similarly, patients' genotyping for MMR mutations is also recommended in stage II CRC. Regarding toxicity, individuals who are candidates for therapy with irinotecan should be tested for UGT1A1 polymorphisms.

Besides these, several other molecules have been studied as potential biomarkers for chemotherapy sensitivity/resistance. However, they are not used in practice, as they have not yet proven their clinical value. Therefore, further investigation should be addressed in order to explore the potential role of these promising markers.

## Figures and Tables

**Table 1:** Summary of potential predictive biomarkers, available data, and current recommendations.

Drug	Potential biomarkers	Data available	Current recommendations
5-FU	TS expression	Inconsistent results	None
	TP expression	Inconsistent results; high level potential indicator of poor prognosis	None
	DPD expression	Not predictive; low levels associated with better prognosis	None

Irinotecan	UGT1A1^*∗*^28/UGT1A1^*∗*^6 polymorphisms	Not predictive; associated with severe toxicity	Patient screening for UGT1A1^*∗*^28 and UGT1A1^*∗*^6 (in Japan) polymorphisms

Oxaliplatin	ERCC1	Inconsistent results	None

Cetuximab/panitumumab	KRAS mutation	Resistance to cetuximab/panitumumab	Patient screening for exon-2 KRAS mutations
	BRAF V600E	Inconsistent results; poor prognosis	BRAF genotyping for stage IV CRC
	PTEN loss	Not predictive	None
	PIK3CA mutation	Not predictive	None

Bevacizumab	VEGF expression	Not predictive; contradictory data	None

	MSI-H	Resistance to chemotherapy; good prognosis	Determination of MSI status for all stage II CRC patients

5-FU: 5-fluorouracil; TS: thymidylate synthase; TP: thymidine phosphorylase; DPD: dihydropyrimidine dehydrogenase; UGT1A1: uridine diphosphate glucuronosyltransferase 1A1; ERCC1: excision repair cross-complementation group 1; VEGF: vascular endothelial growth factor; MSI-H: microsatellite instability-high.
